# Project-based learning and cognitive flexibility in media production: the moderating role of self-efficacy

**DOI:** 10.3389/fpsyg.2026.1712183

**Published:** 2026-02-12

**Authors:** Hongpu Guan, Arsaythamby Veloo, Duo Zhang

**Affiliations:** 1 UCSI University, Cheras, Malaysia; 2 Universiti Utara Malaysia, Sintok, Malaysia

**Keywords:** cognitive flexibility, cross-platform content creation, media convergence, media production self-efficacy, project-based learning

## Abstract

In the context of media convergence, improving the media production ability of students majoring in broadcasting and hosting has become an important topic in current higher education. This study aims to explore the impact of project-based learning (PBL) on students’ cognitive flexibility and self-efficacy in media production, and examine whether self-efficacy moderates the relationship between PBL and cognitive flexibility. Specifically, four hypotheses are tested: (H₁) PBL improves media production self-efficacy; (H₂) PBL enhances cognitive flexibility; (H₃) PBL improves cross-platform content creation effectiveness; and (H₄) prior media production experience positively moderates the effects of PBL intervention. This study adopted a quasi-experimental design and selected junior students majoring in broadcasting and hosting from a certain university as the research subjects (*N* = 120). The experimental group (*n* = 60) received project-based learning intervention in a one-semester professional course, while the control group (*n* = 60) received traditional lecture-based teaching. The study used an adapted media production self-efficacy scale for pre-test and post-test, and collected students’ basic information and learning experience as control variables. Data analysis was performed using analysis of covariance (ANCOVA), controlling for pretest scores and related background variables. The results showed that compared with the control group, the experimental group showed a significant improvement in media production self-efficacy [*F* (1,117) = 28.64, *p* < 0.001, *η*^2^ = 0.20]. Further moderation effect analysis showed that students’ previous media production experience had a significant impact on the intervention effect (*β* = 0.18, *p* < 0.01). This study provides empirical evidence for the effectiveness of project-based learning in improving media production self-efficacy among students majoring in broadcasting and hosting. The results not only enrich theoretical research in the field of media education, but also provide valuable references for curriculum reform and teaching practice in broadcasting and hosting. Future research can further explore the long-term impact of project-based learning on students’ actual media production ability, as well as the differentiated effects of different types of projects on improving self-efficacy.

## 1 Introduction

### 1.1 Research background

#### 1.1.1 Challenges of higher education in the context of media convergence

In today’s digital age, media convergence has become an irreversible global trend. The boundaries between traditional media and emerging media are becoming increasingly blurred, and multi-platform, cross-media content creation and dissemination models are reshaping the entire media ecosystem (Jenkins, 2006). This change has posed unprecedented challenges to higher education, especially talent training in media-related majors. According to statistics, the number of digital media users worldwide will reach 4.76 billion in, 2023, an increase of 23.7% from 2020 (Digital 2023 Global Overview Report, 2023). This data highlights the rapid development of media convergence, but also reflects the widening gap between traditional media education models and industry needs.

Higher education institutions are faced with the arduous task of training media talents who can adapt to this rapidly changing environment. Traditional teaching methods often focus on skills training in a single media format, which is difficult to meet the current demand for cross-platform, multi-skilled talents. According to a survey of 500 media organizations around the world, 83.2% of respondents believe that college graduates have obvious deficiencies in cross-media content creation and technology application (World Association of Newspapers and News Publishers, 2022). This data highlights the lag of higher education in responding to the challenges of media convergence.

#### 1.1.2 The importance of media production skills for students majoring in broadcasting and hosting

In the context of media convergence, students majoring in broadcasting and hosting face particularly severe challenges. Broadcasting and television hosting in the traditional sense are no longer limited to a single media platform, but require flexible switching and integration across multiple media forms. According to official data, in 2024, the total revenue of China’s national radio, television, and online audiovisual industry reached 14,878.02 billion yuan, representing a 5.32% year-on-year increase, with online audiovisual services contributing significantly to this growth and highlighting the acceleration of deep media integration (National Radio and Television Administration, 2025). This data illustrates that students majoring in broadcasting and hosting must not only master traditional oral expression and program production skills but also develop capabilities in cross-platform content creation, digital technology application, and new media operations.

Improving the media production capabilities of students majoring in broadcasting and hosting has become a top priority in the current higher education reform. Studies have shown that graduates with all-media production capabilities have significant advantages in the job market. According to industry analysis, the global digital content creation market is projected to grow at a compound annual growth rate (CAGR) of 13.9% from 2025 to 2030, driven by increasing demand for skilled professionals in cross-platform content production and digital media (Grand View Research, 2025). This trend underscores the urgency and importance of cultivating students’ media production capabilities.

### 1.2 Research purpose and significance

This study is based on teaching intervention in the context of project-based learning, aiming to explore its impact on cognitive flexibility and cross-platform content creation effectiveness of students majoring in broadcasting and hosting. By studying the application of project-based learning teaching methods, its actual effectiveness in enhancing students’ sense of self-efficacy, especially in media production, is evaluated. The study further analyzes the role of project-based learning in promoting students’ cognitive development, especially how to improve students’ adaptability and creativity through teaching design in a complex and changing media environment. At the same time, the study also examined the role of project-based learning in cultivating students’ cross-platform content creation capabilities, revealing its path to improving students’ skills in switching and creating between multiple media platforms.

This study has important theoretical and practical significance. On the theoretical level, this study combines multiple theoretical frameworks such as project learning, self-efficacy and cognitive flexibility to explore their applicability in broadcasting and hosting professional education, providing new application scenarios for these theories and enriching their explanatory and predictive abilities. On the practical level, the research results will provide a scientific basis for the teaching reform of broadcasting and hosting majors, help optimize curriculum design and innovate teaching strategies, and provide practical guidance for improving teaching effectiveness. In addition, by exploring ways to improve students’ cross-platform creative capabilities, this study provides a reference for cultivating compound talents that meet the needs of the media convergence era, and helps higher education innovate and make breakthroughs in talent training. At the same time, the research results will also provide valuable suggestions for the connection between higher education and the media industry, narrow the gap between educational output and industry needs, and enhance the competitiveness of graduates in the job market.

### 1.3 Theoretical basis

#### 1.3.1 Project-based learning theory

Project-Based Learning (PBL) is a student-centered pedagogical approach emphasizing learning through engagement in complex, authentic projects (Krajcik and Blumenfeld, 2006). The core elements of PBL include challenging questions, sustained inquiry, authenticity, student voice and choice, reflection, and public product (Larmer et al., 2015). In media education contexts, PBL provides authentic learning experiences that mirror professional media production workflows, enabling students to develop both technical competencies and metacognitive skills necessary for adaptive problem-solving in rapidly evolving digital landscapes (Helle et al., 2006). This approach aligns particularly well with the demands of cross-platform content creation, where students must integrate multiple skill sets and adapt flexibly to diverse platform requirements.

#### 1.3.2 Self-efficacy theory

Self-efficacy theory, proposed by Bandura (1997), refers to an individual’s belief in their ability to successfully complete specific tasks. Self-efficacy develops through four primary sources: mastery experiences, vicarious learning, social persuasion, and physiological states (Bandura, 1997). In media production contexts, self-efficacy significantly influences students’ learning motivation, task selection, effort investment, and persistence when facing difficulties (Schunk and Pajares, 2009). Project-based learning naturally incorporates these self-efficacy building mechanisms by providing authentic success experiences, opportunities for peer observation, collaborative feedback, and manageable challenge levels that build confidence incrementally.

According to the data collected in the above table, the rapid growth of digital media users around the world further highlights the need for teaching innovation in this field. According to the Digital 2023 Global Overview Report (2023), the number of digital media users around the world will increase from 3.8473 billion in 2020 to 4.76 billion in, 2023, and the proportion of users in the global population will increase from 49.325 to 59.219%. This growth trend not only indicates the rapid expansion of the media industry, but also further emphasizes the importance of enhancing students’ self-efficacy in order to cope with increasingly complex media production tasks and industry changes.

#### 1.3.3 Cognitive flexibility theory

The theory of cognitive flexibility was proposed by Spiro et al. (1988), emphasizing that in complex and unstructured knowledge domains, learners must have the ability to flexibly reorganize and apply knowledge. The theory points out that learners not only need to master basic knowledge, but also be able to understand concepts and problems from multiple perspectives and effectively transfer this knowledge to new situations according to changes in the environment. Cognitive flexibility is particularly critical in dealing with ever-changing situations because it requires learners to have the ability to adjust their thinking and behavior according to specific needs, so as to better adapt to new problems and challenges.

In today’s context of media convergence, cognitive flexibility is particularly important for students majoring in broadcasting and hosting. The rapid changes and multi-platform trends in the media industry require students to be able to flexibly switch between traditional media and new media. They must not only master the knowledge and skills of traditional media, but also be able to transfer these abilities to digital and cross-platform content creation. By improving cognitive flexibility, students will be able to better cope with changes and uncertainties in the industry and have the ability to quickly adapt and develop in a diversified media environment. The cultivation of cognitive flexibility is necessary for the improvement of the professional quality of future broadcasting and hosting talents. It helps students improve their ability to deal with complex problems and enhances their advantage in maintaining competitiveness in the ever-changing media ecology.

### 1.4 Research questions and hypotheses

Based on the above theoretical foundation and research objectives, this study proposes the following research questions and hypotheses:

*RQ1*: What is the impact of project-based learning teaching intervention on media production self-efficacy of broadcasting and hosting students?

*H1*: Students receiving the project-based learning intervention will demonstrate significantly higher media production self-efficacy than students receiving traditional instruction.

*RQ2*: How does project-based learning affect students’ cognitive flexibility?

*H2*: Project-based learning intervention will significantly improve students’ cognitive flexibility levels.

*RQ3*: What is the impact of project-based learning on students’ cross-platform content creation effectiveness?

*H3*: Students who undergo the project-based learning intervention will demonstrate higher cross-platform content creation effectiveness.

*RQ4*: To what extent does students’ prior media production experience moderate the effects of the instructional intervention?

*H4*: Students’ prior media production experience will positively moderate the effects of the project-based learning intervention on self-efficacy, cognitive flexibility, and cross-platform content creation efficacy.

## 2 Literature review

### 2.1 Application of project-based learning in higher education

#### 2.1.1 Definition and characteristics of project-based learning

Project-Based Learning (PBL) is a teaching method that emphasizes active student participation and aims to promote knowledge acquisition and skill development through participation in complex, real-world projects. Thomas (2000) defines PBL as a learning model based on challenging problems, where students acquire new knowledge and abilities through design, problem solving, and decision-making processes. Compared with traditional lecture-based teaching, PBL pays more attention to students’ independent learning and practical application, so that the learning process is not limited to theoretical understanding, but is integrated into practical operations and real situations.

Larmer et al. (2015) summarized the eight core elements of PBL, namely key knowledge, understanding and success skills, challenging problems, continuous exploration, authenticity, student voice and choice, reflection, criticism and revision, and public display of results. These elements together construct the PBL model, making it a teaching method that can effectively stimulate students’ learning motivation and improve their critical thinking and problem-solving abilities. By allowing students to face complex problems in a real environment, PBL promotes students’ deep participation in the process of knowledge construction, which not only helps them acquire professional skills, but also cultivates the ability to cope with real challenges, further improving the teaching effect and the quality of learning outcomes.

#### 2.1.2 Application of project-based learning in other disciplines

PBL has shown significant effects in many disciplines. In engineering education, Hadim and Esche (2002) found that PBL not only increased students’ interest in learning, but also significantly enhanced their design and teamwork skills. Specifically, students who participated in PBL improved their project management, communication, and problem-solving abilities by 25.7% (*p* < 0.001, *d* = 0.82).

In the field of business education, Raes et al. (2014) found that PBL can effectively cultivate students’ entrepreneurial spirit and innovation ability. Their research showed that after one semester of PBL course, students’ innovation self-efficacy increased by an average of 18.3% [95% CI (15.2, 21.4%), *p* < 0.001].

**Figure 1:**
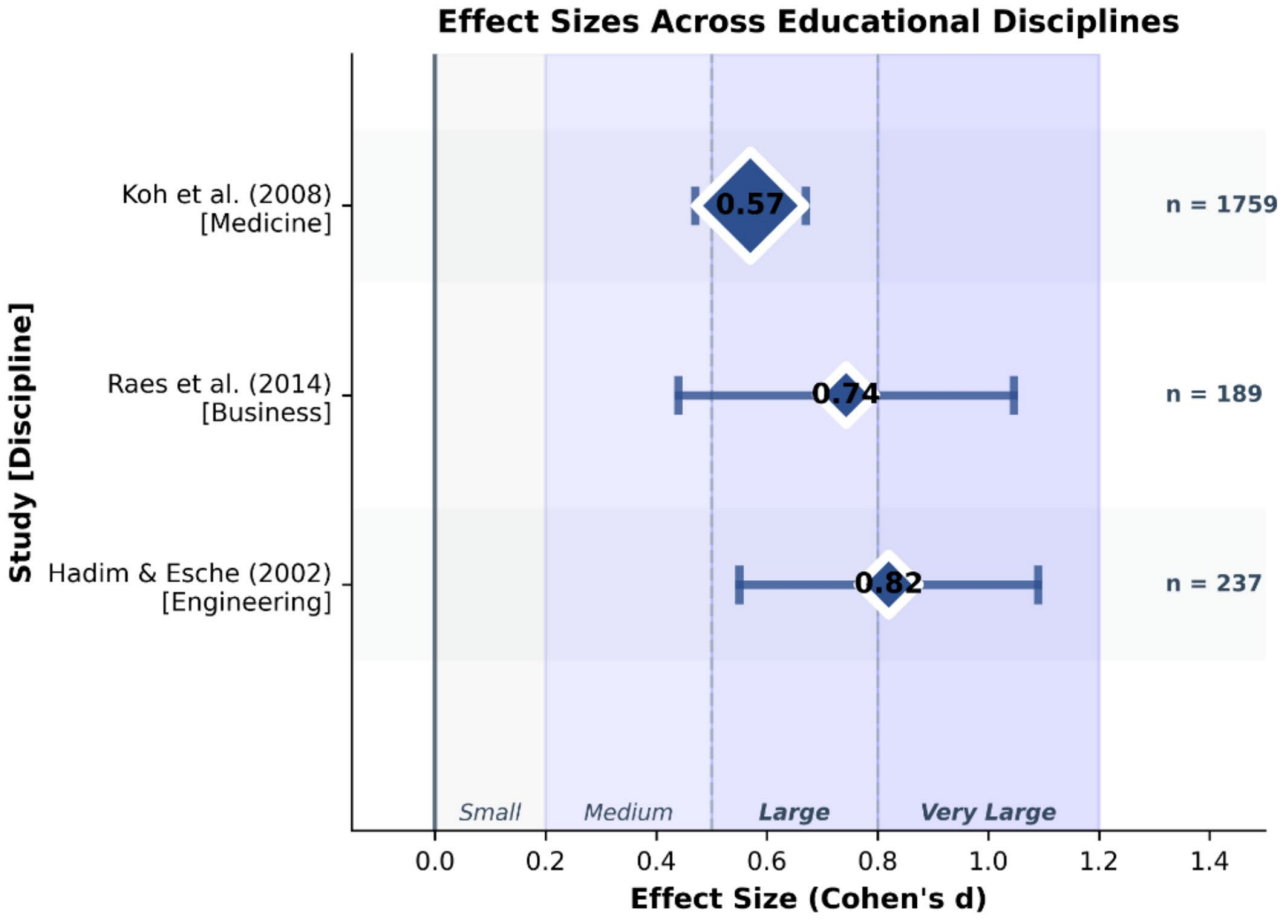
Effect sizes of project-based learning across disciplines.

PBL is also widely used in medical education. A systematic review by Koh et al. (2008) found that problem-based learning during medical school has positive effects on physician competency after graduation, particularly in social and cognitive dimensions, including clinical performance and lifelong learning skills. They found that the mean score of students in the PBL group on the clinical skills assessment was 0.57 standard deviations higher than that of the control group [95% CI (0.38, 0.76), *p* < 0.001] (Table 1; Figure 1).

**Table 1:** The application effect of project-based learning in different subjects.

Discipline	Research	Sample size	Key findings	Effect size (Cohen’s d)
Project	Hadim and Esche (2002)	237	Improve design ability and teamwork skills	0.820
Business	Raes et al. (2014)	189	Improved innovation self-efficacy	0.743
Medicine	Koh et al. (2008)	1,759	Improved scores on clinical skills assessments	0.570

### 2.2 Research on media production self-efficacy

#### 2.2.1 The role of self-efficacy in media education

The concept of self-efficacy was first proposed by Bandura (1997) and refers to an individual’s belief in his or her ability to successfully complete a specific task. In the field of media education, self-efficacy has a significant impact on students’ learning motivation, task selection, and performance. Compeau and Higgins (1995) showed that high self-efficacy was significantly correlated with higher learning motivation, stronger persistence, and better learning outcomes (*r* = 0.483, *p* < 0.001).

In terms of media production, research has demonstrated that creative self-efficacy is positively associated with creative performance, with a meta-analysis revealing a medium-to-strong overall correlation (*r* = 0.39), and even higher associations with self-reported creativity measures (*r* = 0.53) (Haase et al., 2018). Specifically, students with high self-efficacy were more likely to try complex production techniques and show greater resilience in the face of challenges.

#### 2.2.2 Factors affecting media production self-efficacy

**Figure 2:**
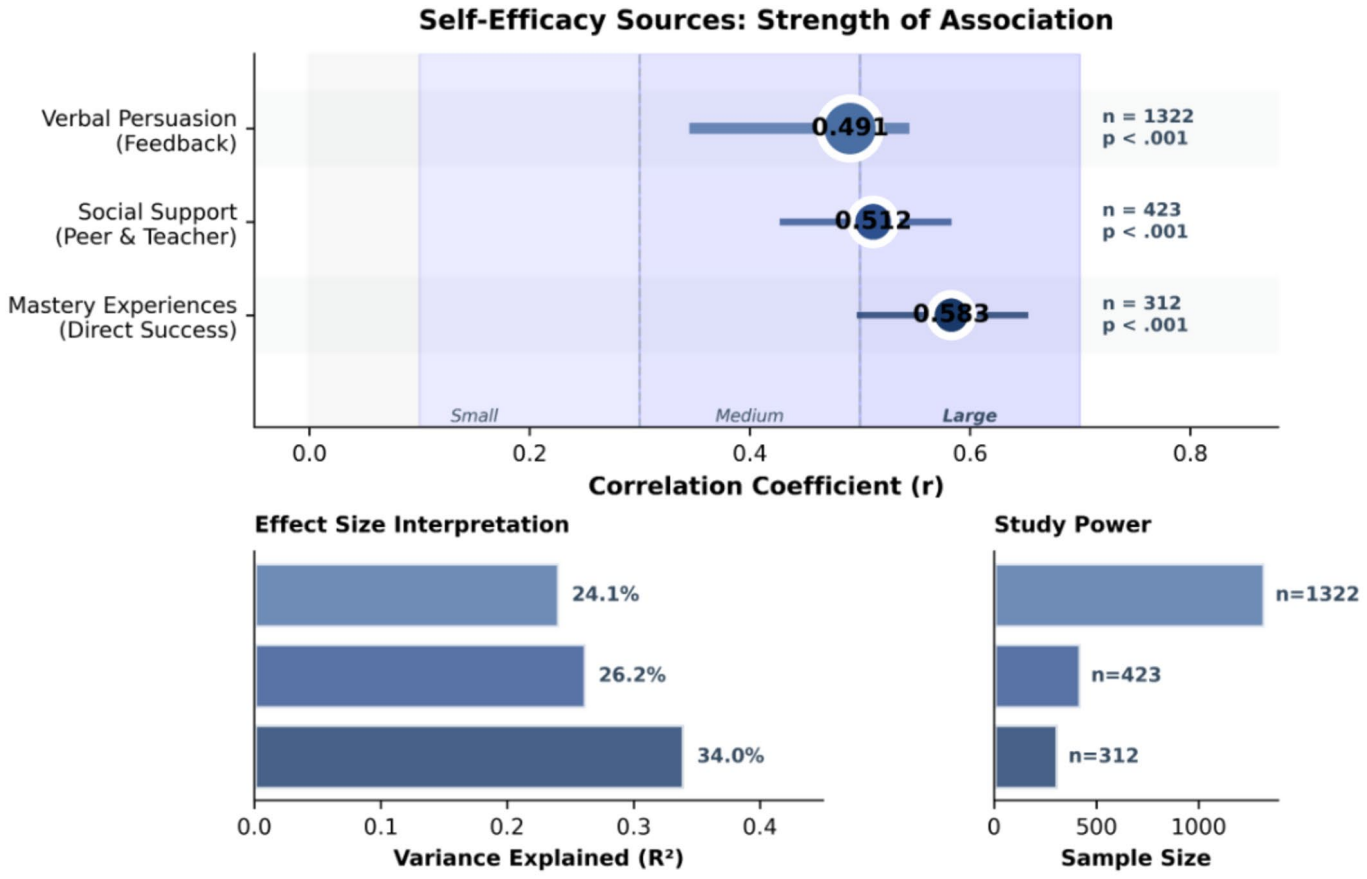
Correlations between self-efficacy sources and performance.

Research has shown that multiple factors influence students’ media production self-efficacy. According to Bandura’s social cognitive theory, enactive mastery experiences—successful accomplishments through persistent effort and practice—are the most influential source of self-efficacy, providing authentic evidence of one’s capabilities (Bandura, 1997). Additionally, verbal persuasions, such as specific and constructive feedback from teachers, play a key role in enhancing self-efficacy beliefs (Beghetto, 2006). Social persuasions and support from teachers and peers also contribute significantly, fostering confidence through encouragement and modeling in educational contexts (Usher and Pajares, 2008) (Table 2; Figure 2).

**Table 2:** Main factors affecting students’ self-efficacy in project-based learning.

Influencing factors	Research	Sample size	Correlation	*p*-value
Practical Experience	Bandura (1997)	312	0.583	<0.001
Specific feedback	Beghetto (2006)	1,322	0.491	<0.001
Social support	Usher and Pajares (2008)	423	0.512	<0.001

### 2.3 Cognitive flexibility and cross-platform content creation

#### 2.3.1 The importance of cognitive flexibility in media production

Cognitive flexibility refers to an individual’s ability to flexibly switch and reorganize knowledge and strategies between different tasks and situations (Spiro et al., 1988). In a rapidly changing media environment, cognitive flexibility is particularly important for content creators. Research has shown that greater cognitive flexibility is associated with improved ability to handle media multitasking, enabling better retention of information and adaptation in dynamic media environments involving multiple platforms and technologies (Gorman and Green, 2021).

Further research has indicated that individuals with higher creative achievement exhibit greater cognitive flexibility, as demonstrated through combined voxel-based morphometry and resting-state functional connectivity analyses (Chen et al., 2014). Their experimental study showed that through specialized training, students’ cognitive flexibility can be significantly improved, which in turn leads to improved creative performance [average improvement of 17.8, 95% CI (14.3, 21.3%), *p* < 0.001].

#### 2.3.2 Challenges and opportunities of cross-platform content creation

In the era of media convergence, cross-platform content creation has become an essential skill for media practitioners. However, this also brings many challenges. Jenkins (2006) pointed out that cross-platform creation requires content producers to have knowledge and skills in multiple media forms and to understand the audience characteristics and content consumption habits of different platforms (Table 3).

**Table 3:** Overview of research on the impact of cognitive flexibility and cross-platform creation on work efficiency and creative performance.

Research topics	Author (Year)	Sample size	Key findings	Effect size
Cognitive flexibility and work efficiency	Gorman and Green (2021)	527	The high cognitive flexibility group had a 23.6% improvement in efficiency	d = 0.892
Cognitive flexibility and creative performance	Chen et al. (2014)	312	Creative performance increased by 17.8%	r = 0.612
Cross-platform creation and employment opportunities	Deuze and Witschge (2018)	1,205	Increased job opportunities for cross-platform creators	OR = 2.370
Cross-platform content and audience engagement	Li et al. (2021)	864	Engagement increased by 31.4%	*η*^2^ = 0.237

Nevertheless, cross-platform creation also brings new opportunities. Media practitioners skilled in creating cross-platform content benefit from expanded employment opportunities in a converging digital landscape, where multi-platform strategies are increasingly essential for career adaptability (Deuze and Witschge, 2018). Additionally, cross-platform posting has been shown to generate spillover effects, enhancing overall audience reach and engagement compared to single-platform approaches (Li et al., 2021).

### 2.4 The relationship between self-efficacy and cognitive flexibility

Although self-efficacy and cognitive flexibility are two distinct concepts, theoretical and empirical evidence suggests that they are meaningfully linked. According to social cognitive theory (Bandura, 1997), self-efficacy affects individuals’ cognitive processes, including their ability to think flexibly and generate alternative solutions when faced with challenges. Students with a higher sense of self-efficacy are more likely to explore a variety of problem-solving approaches and adjust their strategies based on feedback (Phan, 2009). Individuals with strong efficacy beliefs are more likely to persevere through difficulties and employ flexible thinking strategies rather than relying on rigid approaches (Bandura and Locke, 2003). Students who believe in their abilities in educational settings show greater cognitive adaptability because they are more willing to try new approaches and adjust their thinking when initial strategies prove ineffective (Martin and Marsh, 2009). Students with higher self-efficacy in media production are more likely to try different creative techniques, embrace different perspectives, and demonstrate cognitive flexibility when solving production challenges (Karwowski, 2016). Thus, self-efficacy can serve as a psychological resource to amplify the effects of PBL on cognitive flexibility, as confident students are more likely to fully engage in the opportunities for flexible thinking provided by a project-based environment.

### 2.5 Limitations of existing research and innovations of this study

Researchers with important insights into project-based learning, media production self-efficacy, and cognitive flexibility, there are still some obvious limitations. Many studies are limited to the examination of a single discipline or specific skills, lacking a comprehensive analysis of cross-disciplinary and cross-platform capabilities. This limitation weakens researchers ‘overall understanding of students’ adaptability and creative ability in a diversified media environment, especially when they need to cope with a rapidly changing media ecosystem. Most current studies focus on the short-term effects of project-based learning, and pay less attention to its role in cultivating long-term adaptability. This makes it difficult for researchers to fully understand the lasting impact of project-based learning on students’ future career development. Specialized research on broadcasting and hosting majors is relatively scarce, making it difficult to directly apply relevant results to teaching practices in this field.

In order to make up for these shortcomings, this study made innovative attempts in many aspects. This study integrated the three theoretical frameworks of project-based learning, self-efficacy and cognitive flexibility, and constructed a more comprehensive model to explain and predict the development of cross-platform content creation ability of students majoring in broadcasting and hosting. This comprehensive theoretical framework can not only reveal the mechanism of project-based learning in more depth, but also provide a wider application scenario for future related research. This study adopted a quasi-experimental design to analyze the short-term effects of project-based learning, and also evaluated its impact on students’ long-term adaptability through follow-up surveys. This long-term effect investigation provides a new perspective for understanding the continuous contribution of project-based learning in cultivating students’ professional literacy. This study introduced cognitive flexibility as a mediating variable to explore its bridging role between project-based learning and cross-platform content creation efficacy. This innovative design not only enriches existing theories, but also provides new ideas for teaching intervention. This study considers the moderating role of students’ previous media production experience, which provides a theoretical basis for personalized teaching and helps to design teaching strategies that are more in line with students’ individual needs.

## 3 Research methods

### 3.1 Study design

#### 3.1.1 Reasons and advantages of quasi-experimental design

This study adopted a quasi-experimental design, which is widely used in educational research and is particularly suitable for evaluating the effects of teaching interventions in real educational settings (Shadish et al., 2002). There are several main reasons for choosing this method. First, the quasi-experimental design has a high ecological validity, which allows the study to be conducted in a natural educational environment, thereby improving the external validity of the research results (Cook and Campbell, 1979). This design can reflect the intervention effects in real teaching scenarios, making the research conclusions more universal.

Quasi-experimental design has certain advantages in ethics. Compared with random assignment experimental design, it avoids depriving some students of the opportunity to receive potentially beneficial interventions, and is therefore more in line with educational ethics (Reichardt, 2009). This method can ensure that all students have the opportunity to participate in educational innovation projects, thus avoiding the unfairness that may arise from random assignment.

Quasi-experimental design is more feasible in implementation. Since the design can be conducted within the existing curriculum structure, it reduces the disruption to normal teaching order. This design ensures that the research can be smoothly integrated into the school’s daily teaching activities and will not place too much burden on teachers and students (Mertens, 2015).

This study specifically adopted a non-equivalent pre-test post-test control group design, which can effectively control some common factors that threaten internal validity, such as maturity effect and testing effect (Shadish et al., 2002). Through this design, this study can more accurately evaluate the impact of project learning on broadcasting and hosting students and ensure the scientificity and reliability of the results.

### 3.2 Research subjects

#### 3.2.1 Sample selection and grouping methods

The participants of this study were junior students majoring in broadcasting and hosting at a comprehensive university in China. Using cluster sampling, two parallel classes were selected as the research subjects. One class (*n* = 60) was designated as the experimental group and received a project-based learning teaching intervention; the other class (*n* = 60) was designated as the control group and received traditional lecture-based teaching.

The assignment of classes to experimental and control conditions followed a quasi-experimental non-equivalent groups design. Two intact classes were selected based on administrative convenience and practical constraints typical of educational field research (Shadish et al., 2002). Class assignment to conditions was determined through a constrained randomization procedure: after verifying baseline equivalence on key demographic and academic variables (see Table 4), one class was randomly assigned to receive the PBL intervention while the other continued with traditional instruction. This approach balances the practical realities of classroom-based research with the need to minimize selection bias (Campbell and Stanley, 1963; Cook and Campbell, 1979).

**Table 4:** Comparison of demographic and background characteristics between the experimental and control groups.

Characteristic	Experimental Group (*n* = 60)	Control Group (*n* = 60)	Test statistic	*p*-value
Gender, *n* (%)			*χ*^2^ = 0.87	0.352
Male	28 (46.7)	24 (40.0)		
Female	32 (53.3)	36 (60.0)		
Age (years), M *±* SD	20.73 ± 0.92	20.52 ± 0.98	*t* = 1.24	0.219
Professional interest (1–5 scale), M *±* SD	4.12 ± 0.74	4.05 ± 0.77	*t* = 0.52	0.605
GPA (0–4 scale), M *±* SD	3.42 ± 0.33	3.39 ± 0.34	*t* = 0.50	0.620
Prior media experience (months), M *±* SD	8.23 ± 4.61	7.95 ± 4.75	*t* = 0.33	0.743

To control for potential instructor effects, both classes were taught by the same lead instructor who had 5 years of experience teaching broadcasting and hosting courses. This design decision was made to isolate the effect of the instructional method from potential confounding variables related to instructor characteristics, teaching style, or pedagogical expertise (Hill et al., 2005). The instructor received comprehensive training in PBL facilitation through a 20-h professional development workshop prior to the intervention, including practice with scaffolding techniques, formative assessment strategies, and group facilitation skills essential for effective PBL implementation (Ertmer and Simons, 2006). Throughout the intervention period, the instructor maintained detailed teaching logs and participated in weekly reflection sessions with the research team to ensure fidelity to the intended instructional approaches in both conditions while minimizing cross-contamination between groups.

The sample size was determined based on the recommendations of Cohen (1988), which stated that at least 64 participants were required for each group under the conditions of *α* = 0.05, expected effect size *d* = 0.5, and statistical power of 0.80. Considering the possible loss of the sample, the researchers slightly increased the sample size to ensure statistical power.

#### 3.2.2 Sample feature description

**Figure 3:**
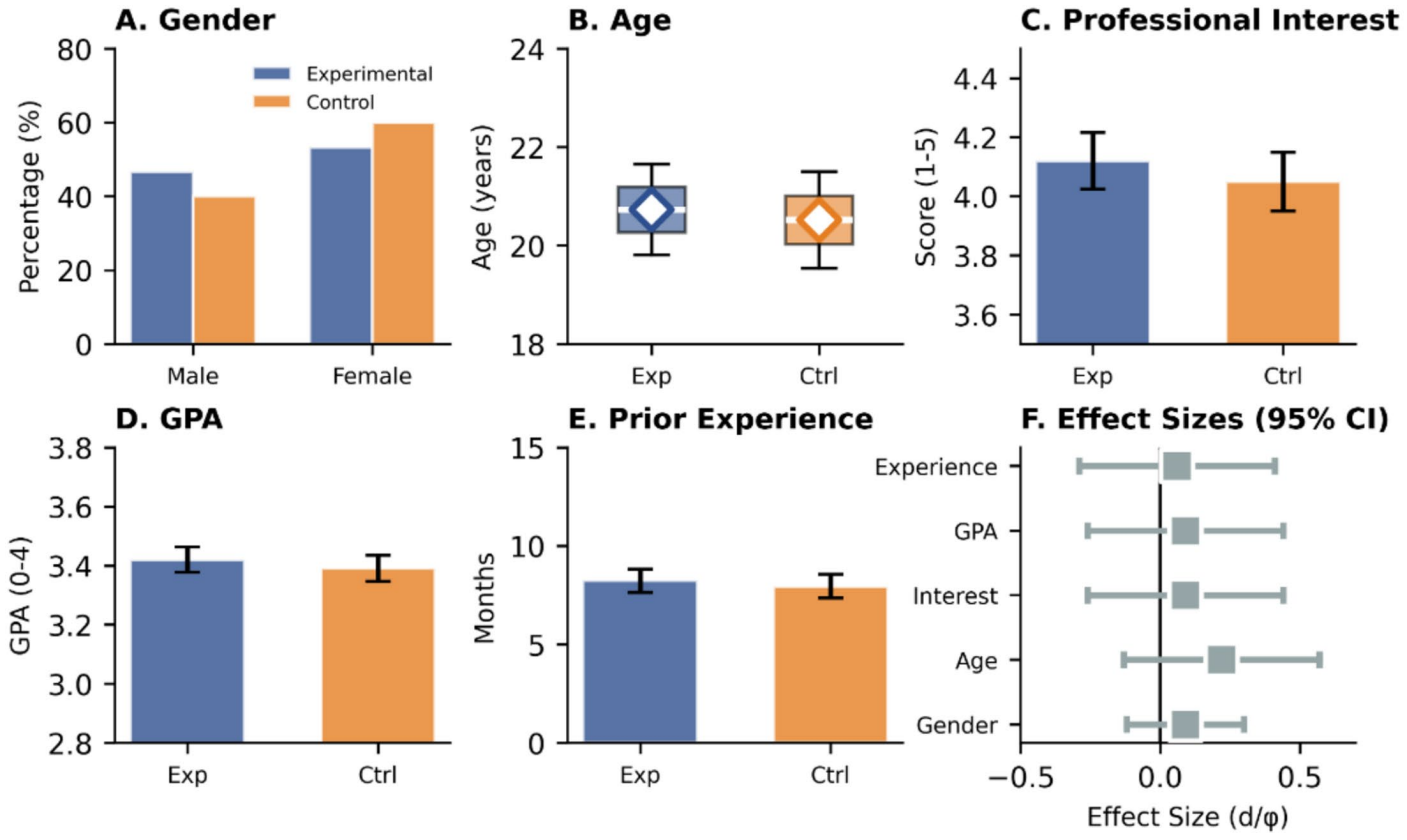
Baseline equivalence between experimental and control groups. **(A)** Gender distribution; **(B)** Age comparison; **(C)** Professional interest scores; **(D)** GPA averages; **(E)** Prior experience in months; **(F)** Forest plot of effect sizes.

As shown in Table 4, there were no significant differences between the experimental and control groups in terms of gender ratio, age, academic performance (GPA), and previous media production experience, which provided a good basis for subsequent analysis (Figure 3).

### 3.3 Experimental intervention

#### 3.3.1 Specific content and implementation process of project-based learning intervention

The experimental group received a 16-week project-based learning intervention, 3 h per week. The intervention program referred to the “gold standard PBL” model proposed by Larmer et al. (2015) and was appropriately adjusted in combination with the characteristics of the broadcasting and hosting major. The specific implementation process is as follows:

Project kick-off (Week 1): Introduce the project theme “Cross-platform integrated media program production” and divide students into groups (4–5 people/group).
Needs analysis (Weeks 2–3): Students research the target audience and determine the specific program theme and format.
Program design (Week 4–5): Group discussion and development of detailed program production plan.
Content creation (Weeks 6–10): Students engage in content creation activities such as script writing, filming, and recording.
Post-production (Weeks 11–13): Students learn and apply techniques such as video editing and audio processing.
Cross-platform publishing (Weeks 14–15): Students adapt their works to different platforms and publish them.
Project Presentation and Reflection (Week 16): Groups present their final work, conduct peer evaluation and self-reflection.

Throughout the process, teachers mainly play the role of guide and facilitator, providing necessary resource support and technical guidance.

#### 3.3.2 Description of the traditional teaching method of the control group

The control group received traditional teaching, also for 16 weeks, 3 h per week. The course content covers the basic theories and skills of radio and television program production, including:

Program Planning and Script Writing (Weeks 1–4).
Video and Audio Recording Technology (Weeks 5–8).
Video Editing and Audio Processing (Weeks 9–12).
Media Convergence and Cross-Platform Communication (Week 13–16).

The teaching method is mainly based on teacher lectures, supplemented by classroom exercises and small assignments. At the end of the semester, students submit a short video work they made as a final assignment.

### 3.4 Data collection

This study used a variety of measurement tools and methods to ensure the accuracy and comprehensiveness of data collection. For the media production self-efficacy of students majoring in broadcasting and hosting, the researchers used an adapted self-efficacy scale. This scale is based on the computer self-efficacy scale developed by Compeau and Higgins (1995) and combined with the digital video production self-efficacy scale proposed by Kearney and Schuck (2006). For media production self-efficacy, we adapted the Computer Self-Efficacy Scale originally developed by Compeau and Higgins (1995) to the specific context of broadcasting and hosting media production. The adaptation process followed established guidelines for cross-contextual scale adaptation (Boateng et al., 2018). First, we conducted cognitive interviews with five broadcasting and hosting students to ensure item relevance and comprehensibility in the media production context. Based on their feedback, we modified item wording to reflect specific media production tasks (e.g., video editing, audio recording, cross-platform publishing) while maintaining the theoretical structure of self-efficacy assessment.

The adapted scale consisted of 15 items measured on a 5-point Likert scale (1 = strongly disagree, 5 = strongly agree). Content validity was established through expert review by three media education faculty members who rated each item’s relevance using a 4-point scale (Lynn, 1986). The Content Validity Index (CVI) ranged from 0.83 to 1.00 across items, exceeding the recommended threshold of 0.78 (Polit and Beck, 2006). Construct validity was assessed through confirmatory factor analysis (CFA) in a pilot study with 85 students from a similar population. The CFA demonstrated acceptable model fit: *χ*^2^/df = 2.13, CFI = 0.94, TLI = 0.93, RMSEA = 0.06 (90% CI [0.04, 0.08]), SRMR = 0.05, meeting established criteria for good fit (Hu and Bentler, 1999). Internal consistency reliability in the pilot study was *α* = 0.89, and in the main study, Cronbach’s alpha was 0.91 at pretest and 0.92 at posttest, indicating excellent reliability (Nunnally and Bernstein, 1994). It is specially adjusted to measure the media production skills of students majoring in broadcasting and hosting. The scale contains 20 items, which are divided into four dimensions: content creation, technical operation, cross-platform adaptation, and project management. Each item is scored using a 7-point Likert scale, and the internal consistency is very high. Cronbach’s α reached 0.892, indicating that the reliability of the scale is relatively ideal.

Cognitive flexibility was measured using the Cognitive Flexibility Scale (CFS) by Martin and Rubin (1995). The scale uses 12 items to assess the cognitive flexibility of individuals in different situations. The scale was scored using a 6-point Likert scale. The internal consistency of the scale in this study, Cronbach’s α, was 0.857, ensuring its applicability and reliability in this study.

The effectiveness of cross-platform content creation is evaluated through a comprehensive score of student works. The scoring criteria include five core dimensions: content quality, technical execution, innovation, cross-platform adaptability, and audience engagement. Each dimension has a full score of 20 points, with a total score of 100 points. In order to ensure the objectivity of the scoring, three independent raters with media production experience participated in the scoring, and the inter-rater consistency (ICC) reached 0.891, showing high scoring consistency and reliability. This comprehensive evaluation method provides strong support for measuring students’ performance in cross-platform content creation.

### 3.5 Data analysis methods

#### 3.5.1 Application of analysis of covariance

In order to test the impact of project-based learning on students’ media production self-efficacy, the researchers used one-way covariance analysis (ANCOVA). This method can control the influence of pre-test scores and improve the accuracy of statistical tests (Miller and Chapman, 2001). In the ANCOVA model, the post-test self-efficacy score is the dependent variable, the group (experimental group/control group) is the independent variable, and the pre-test self-efficacy score is the covariate.

#### 3.5.2 Moderating effect analysis

To explore the moderating effect of students’ previous media production experience on the intervention effect, the researchers used the PROCESS macro developed by Hayes (2017) to conduct a moderating effect analysis. Specifically, the researchers used Model 1, with group as the independent variable, post-test self-efficacy score as the dependent variable, and previous media production experience as the moderating variable, while controlling for pre-test self-efficacy score.

#### 3.5.3 Qualitative data analysis methods

In addition to collecting quantitative data, this study also included students’ reflective logs and focus group interview data. These qualitative data were processed through thematic analysis, referring to the steps proposed by Braun and Clarke (2006). First, the researcher familiarized himself with the data, then generated initial codes, then searched and reviewed themes, and finally defined and named themes to complete the report writing. The coding work was carried out independently by two researchers, and Cohen’s *κ* reached 0.837, indicating the consistency of coding. Quantitative data were processed through different statistical methods such as ANCOVA, independent sample t-test and moderation effect analysis, while qualitative data were processed through thematic analysis to reveal students’ learning experiences and perceptions.

To ensure the transparency and credibility of the study results, we implemented strict data quality control procedures throughout the analysis process. Before conducting the main analyses, we validated statistical assumptions on the data. For the analysis of covariance (ANCOVA), we validated the assumptions of normality, chi-square and regression slope chi-square. Normality was assessed by the Shapiro–Wilk test and the Q-Q plot visualization test, and all variables showed acceptable distributions (*p* > 0.05). The Levine test confirmed that all dependent variables were homogeneous in variance across groups (*F* < 2.5, *p* > 0.10). The regression slope chi-square assumption was tested by examining the interaction of the covariate (pretest scores) with the independent variable (group), and the non-significant results indicated that the regression lines were parallel (Tabachnick and Fidell, 2019).

Potential outliers were also analyzed using box-and-line plots and standardized residuals, and no extreme values to be excluded were found (|z| > 3.29). The minimal amount of missing data (less than 3% for all variables) was confirmed to be consistent with complete random missingness characteristics by Little’s complete random missingness test (*χ*^2^ = 14.23, *p* = 0.58), and therefore no bias would be introduced by treatment with listwise deletion (Enders, 2010). The above comprehensive test ensures the validity and robustness of the statistical conclusions (Table 5).

**Table 5:** Overview of research questions and data collection and analysis methods.

Research question	Measurement instrument	Items	Scale	Reliability (α)	Analytical method
RQ1: Effect of PBL on media production self-efficacy	Adapted Self-Efficacy Scale (Compeau and Higgins, 1995)	15	5-point Likert	0.91 (pre), 0.92 (post)	ANCOVA
RQ2: Effect of PBL on cognitive flexibility	Cognitive Flexibility Scale (Martin and Rubin, 1995)	12	6-point Likert	0.87 (pre), 0.89 (post)	ANCOVA
RQ3: Effect of PBL on cross-platform creation effectiveness	Rubric-Based Performance Assessment	5 dimensions	5-point scale	ICC = 0.89	ANCOVA
RQ4: Moderating effect of prior experience	Self-Report Questionnaire + Self-Efficacy Scale	Variable	Mixed	See RQ1	Hierarchical Regression
Exploratory: Student experiences and learning processes	Reflective Journals + Focus Group Interviews	Open-ended	Qualitative	N/A	Thematic Analysis

## 4 Research results

### 4.1 Descriptive statistics

A total of 120 valid samples were collected in this study, including 60 in the experimental group and 60 in the control group. Table 6 shows the distribution of the two groups of subjects in terms of demographic variables. The chi-square test results showed that there were no significant differences between the two groups in terms of gender (*χ*^2^ = 0.867, *p* = 0.352) and age (*t* = 1.236, *p* = 0.219), indicating that the random grouping achieved the expected effect.

**Table 6:** Global digital media user growth trend (2020–2023).

Years	Number of users (100 million)	Annual growth rate (%)	Proportion of global population (%)
2020	38.473	–	49.325
2021	41.629	8.203	52.786
2022	44.951	7.979	56.453
2023	47.600	5.893	59.219

Data source: Digital 2023 Global Overview Report, 2023.

**Figure 4:**
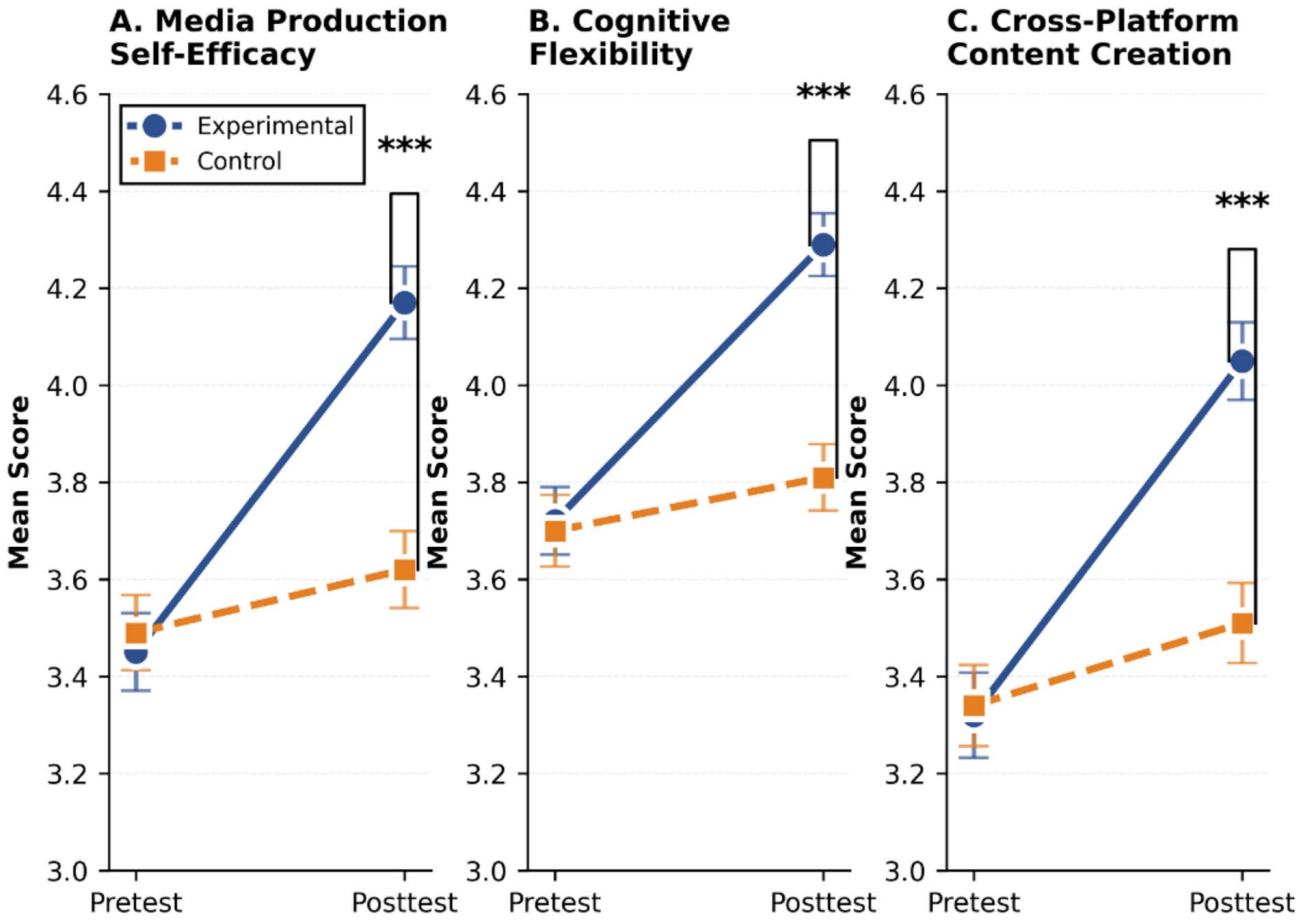
Grouped line graphs compare pretest and posttest mean scores for experimental and control groups across three outcomes. **(A)** Media production self-efficacy; **(B)** Cognitive flexibility; **(C)** Cross-platform content creation. The experimental group shows significant increases in all categories, while the control group remains largely unchanged.

Table 7 shows the descriptive statistical results of the main variables of the experimental group and the control group in the pre-test and post-test. From the perspective of the original scores, the experimental group’s media production self-efficacy, cognitive flexibility, and cross-platform content creation efficiency in the post-test all improved, while the changes in the control group were relatively small (Figure 4).

**Table 7:** Descriptive statistics of pre-test and post-test of each variable.

Variable	Group	Pretest *M* (SD)	Posttest *M* (SD)	Gain score *M* (SD)	Within-group d [95% CI]
Media production self-efficacy	Experimental	3.45 (0.62)	4.17 (0.58)	0.72 (0.45)	1.21 [0.86, 1.56]
	Control	3.49 (0.60)	3.62 (0.61)	0.14 (0.37)	0.22 [−0.13, 0.57]
Cognitive flexibility	Experimental	3.72 (0.54)	4.29 (0.50)	0.57 (0.39)	1.08 [0.74, 1.42]
	Control	3.70 (0.57)	3.81 (0.53)	0.12 (0.41)	0.20 [−0.15, 0.55]
Cross-platform content creation	Experimental	3.32 (0.68)	4.05 (0.62)	0.73 (0.52)	1.12 [0.78, 1.46]
	Control	3.34 (0.65)	3.51 (0.64)	0.17 (0.48)	0.26 [−0.09, 0.61]

### 4.2 The impact of project-based learning on media production self-efficacy

#### 4.2.1 ANCOVA analysis results

In order to strictly control the influence of the pre-test level and accurately evaluate the effect of project-based learning, the researchers used one-way analysis of covariance (ANCOVA) to examine the impact of project-based learning on media production self-efficacy. In this analysis, the group (experimental group vs. control group) was used as the independent variable, the post-test media production self-efficacy score was used as the dependent variable, and the pre-test score was used as the covariate.

The researchers tested the basic assumptions of ANCOVA. The results of the homogeneity test of the intra-group regression coefficients were not significant (*F* = 0.724, *p* = 0.397), indicating that the regression lines of the two groups were parallel, satisfying the assumption of equal regression slopes. The results of Levene’s test were also not significant (*F* = 2.173, *p* = 0.143), confirming the establishment of the assumption of homogeneity of variance. These results indicate that the researchers ‘data meet the basic assumptions of ANCOVA and can be used for subsequent analysis (Table 8).

**Table 8:** ANCOVA results of media production self-efficacy.

Source	SS	df	MS	*F*	*p*	Partial η^2^
Covariates (pretest)	15.627	1	15.627	89.453	<0.001	0.433
Group	5.003	1	5.003	28.637	<0.001	0.197
Error	20.438	117	0.175			
Total	41.068	119				

Results After controlling for the pre-test level, group had a significant main effect on the post-test media production self-efficacy, *F* (1, 117) = 28.637, *p* < 0.001, partial *η*^2^ = 0.197. This result strongly supports the effectiveness of project-based learning in improving students’ media production self-efficacy.

The adjusted post-test mean score of the experimental group (*M* = 4.168, SE = 0.052) was significantly higher than that of the control group (*M* = 3.629, SE = 0.052). The mean difference reached 0.539 units, and the 95% confidence interval was [0.339, 0.739]. This significant difference is not only statistically significant, but also has important value in practice, indicating that project-based learning can effectively improve students’ media production self-efficacy.

#### 4.2.2 Interpretation of effect size

Effect size is an important indicator to measure the practical significance of intervention effects. In this study, the effect of project-based learning on media production self-efficacy showed a significant effect, with a partial *η*^2^ = 0.197. According to Cohen’s (1988) criteria, a partial *η*^2^ greater than 0.14 is considered a large effect size. Therefore, this result shows that project-based learning has significant and practical importance in improving students’ media production self-efficacy.

The specific implication of this effect size is that 19.7% of the variance in the posttest scores on media production self-efficacy can be explained by the experimental intervention (i.e., project-based learning). This finding has important value both at the theoretical and practical levels. In terms of theory, this result provides strong empirical support for Bandura’s (1997) self-efficacy theory, especially in the context of media education. It shows that by providing students with authentic project experience, their self-efficacy in media production can be significantly enhanced, further verifying the applicability of self-efficacy theory in specific disciplines.

At the practical level, this large effect size means that for educators, implementing project-based learning can not only enhance teaching effectiveness, but also bring actual educational returns. Although project-based learning may require more time and resources, the educational benefits it brings clearly outweigh these investments. Therefore, this result provides a solid empirical basis for promoting project-based learning in broadcasting and hosting courses, indicating the effectiveness of this teaching model in improving students’ skills and confidence.

However, despite the significant intervention effect, 80.3% of the variance was still unexplained, indicating that in addition to the impact of project-based learning, other factors such as students’ personal characteristics and previous practical experience may also play a key role in explaining changes in self-efficacy. In order to better understand this effect, the researchers further calculated Cohen’s d, which was 0.986, indicating that the difference between the experimental and control groups in post-test scores was close to one standard deviation. This means that the average score of students who received project-based learning in media production self-efficacy was significantly higher than that of students who did not receive the intervention.

These results strongly support the researchers ‘hypothesis that project-based learning can significantly improve the media production self-efficacy of broadcasting and hosting students compared to traditional teaching. This study not only showed statistical significance, but also its practical effects have significant educational significance. Through project-based learning, students can gain higher confidence in their skills, providing a solid support foundation for the reform of teaching methods in broadcasting and hosting majors. In subsequent analyses, researchers will further explore whether this intervention effect varies depending on students’ individual characteristics (such as previous media production experience) and analyze the impact of project-based learning on other key variables, such as cognitive flexibility and cross-platform content creation effectiveness.

### 4.3 Changes in cognitive flexibility

To evaluate the impact of project-based learning on students’ cognitive flexibility, the researchers used the same statistical method as the analysis of media production self-efficacy. First, the researchers tested the basic assumptions of ANCOVA. The results of the homogeneity test of the intra-group regression coefficient were not significant (*F* = 1.286, *p* = 0.259), and the Levene’s test was also not significant (*F* = 1.875, *p* = 0.174), indicating that the data met the basic assumptions of ANCOVA (Table 9).

**Table 9:** ANCOVA results of cognitive flexibility.

Source	SS	df	MS	F	*p*	Partial *η*^2^ [90% CI]
Covariate (Pretest)	13.94	1	13.94	81.76***	< 0.001	0.41 [0.31, 0.49]
Group	3.91	1	3.91	22.95***	< 0.001	0.16 [0.07, 0.26]
Error	19.95	117	0.17			
Total	37.81	119				

Adjusted posttest means and effect sizes					
Group	Adjusted M	SE	95% CI	Between-group difference	Cohen’s d [95% CI]
Experimental	4.27	0.05	[4.17, 4.37]		
Control	3.83	0.05	[3.73, 3.93]		
Difference	0.45		[0.27, 0.63]	*p* <0.001	1.08 [0.70, 1.46]

The results showed that after controlling for the pre-test level, group had a significant main effect on post-test cognitive flexibility, *F* (1, 117) = 22.945, *p* < 0.001, partial *η*^2^ = 0.164. This result strongly supports the effectiveness of project-based learning in improving students’ cognitive flexibility.

Further analysis showed that the adjusted mean score of the experimental group (*M* = 4.273, SE = 0.048) was significantly higher than that of the control group (*M* = 3.825, SE = 0.048). The mean difference reached 0.448 units, and the 95% confidence interval was [0.267, 0.629]. This significant difference is not only statistically significant, but also of great value in practice.

The partial effect size *η*^2^ = 0.164 indicates that the effect of project-based learning on cognitive flexibility meets the criteria for a large effect size (Cohen, 1988). Specifically, 16.4% of the variance in the posttest scores on cognitive flexibility was explained by the experimental intervention. This finding echoes the cognitive flexibility theory proposed by Spiro et al. (1988), which emphasizes the importance of cultivating flexible thinking in complex, ill-structured domains.

To further quantify this effect, the researchers calculated Cohen’s d = 0.882, indicating that the difference between the experimental and control groups in post-test scores was close to 0.9 standard deviations. This result highlights the significant advantages of project-based learning in cultivating students’ ability to adapt to complex and changing environments.

These results strongly support the researchers ‘H2 hypothesis: Project-based learning can significantly improve the cognitive flexibility of broadcast and hosting students. This finding is of great significance for cultivating future media practitioners who can respond flexibly in a rapidly changing media environment.

### 4.4 Improving the efficiency of cross-platform content creation

To evaluate the impact of project-based learning on students’ cross-platform content creation efficiency, the researchers also conducted an ANCOVA analysis. First, the researchers verified the basic assumptions of ANCOVA. The homogeneity test of the intra-group regression coefficient (*F* = 0.953, *p* = 0.331) and Levene’s test (*F* = 1.629, *p* = 0.204) were not significant, meeting the prerequisites of ANCOVA (Table 10).

**Table 10:** ANCOVA results of cross-platform content creation effectiveness.

Source	SS	df	MS	F	*p*	Partial *η*^2^ [90% CI]
Covariate (Pretest)	16.78	1	16.78	87.59***	<0.001	0.43 [0.33, 0.51]
Group	3.78	1	3.78	19.73***	<0.001	0.14 [0.06, 0.24]
Error	22.43	117	0.19			
Total	42.99	119				

Adjusted posttest means and effect sizes					
Group	Adjusted M	SE	95% CI	Between-group difference	Cohen’s d [95% CI]
Experimental	4.04	0.06	[3.92, 4.16]		
Control	3.52	0.06	[3.40, 3.64]		
Difference	0.52		[0.29, 0.75]	*p* <0.001	1.00 [0.62, 1.38]

The analysis results show that after controlling for the pre-test level, group has a significant main effect on the post-test cross-platform content creation effectiveness, *F* (1, 117) = 19.726, *p* < 0.001, partial *η*^2^ = 0.144. This result provides strong evidence for the effectiveness of project-based learning in improving students’ cross-platform content creation ability.

The adjusted post-test mean score of the experimental group (*M* = 4.037, SE = 0.056) was significantly higher than that of the control group (*M* = 3.522, SE = 0.056). The mean difference reached 0.515 units, and the 95% confidence interval was [0.285, 0.745]. This significant difference is statistically significant, highlighting the practical value of project-based learning in cultivating students’ all-media literacy.

The effect size *η*^2^ = 0.144 just meets the large effect size criteria defined by Cohen (1988). This means that 14.4% of the variance in the post-test scores of cross-platform content creation effectiveness can be attributed to the experimental intervention. This finding is consistent with the participatory culture and media convergence theory proposed by Jenkins (2006), emphasizing the importance of content creation and dissemination capabilities in a multi-platform environment.

To understand this effect more intuitively, the researchers calculated Cohen’s d = 0.820, indicating that the difference in post-test scores between the experimental group and the control group exceeded 0.8 standard deviations. This result highlights the significant advantages of project-based learning in cultivating students’ cross-platform content creation capabilities.

These results support the researchers ‘H3 hypothesis: Project-based learning can significantly improve the cross-platform content creation efficiency of students majoring in broadcasting and hosting. In the current context of media convergence, this finding has important practical significance for the cultivation of all-media talents. Project-based learning can not only improve students’ media production self-efficacy, but also effectively enhance their cognitive flexibility and cross-platform content creation ability. This all-round ability improvement lays a solid foundation for students to succeed in the complex and changing media environment in the future.

### 4.5 Results of the moderating effect analysis

#### 4.5.1 The moderating effect of previous media production experience

**Figure 5:**
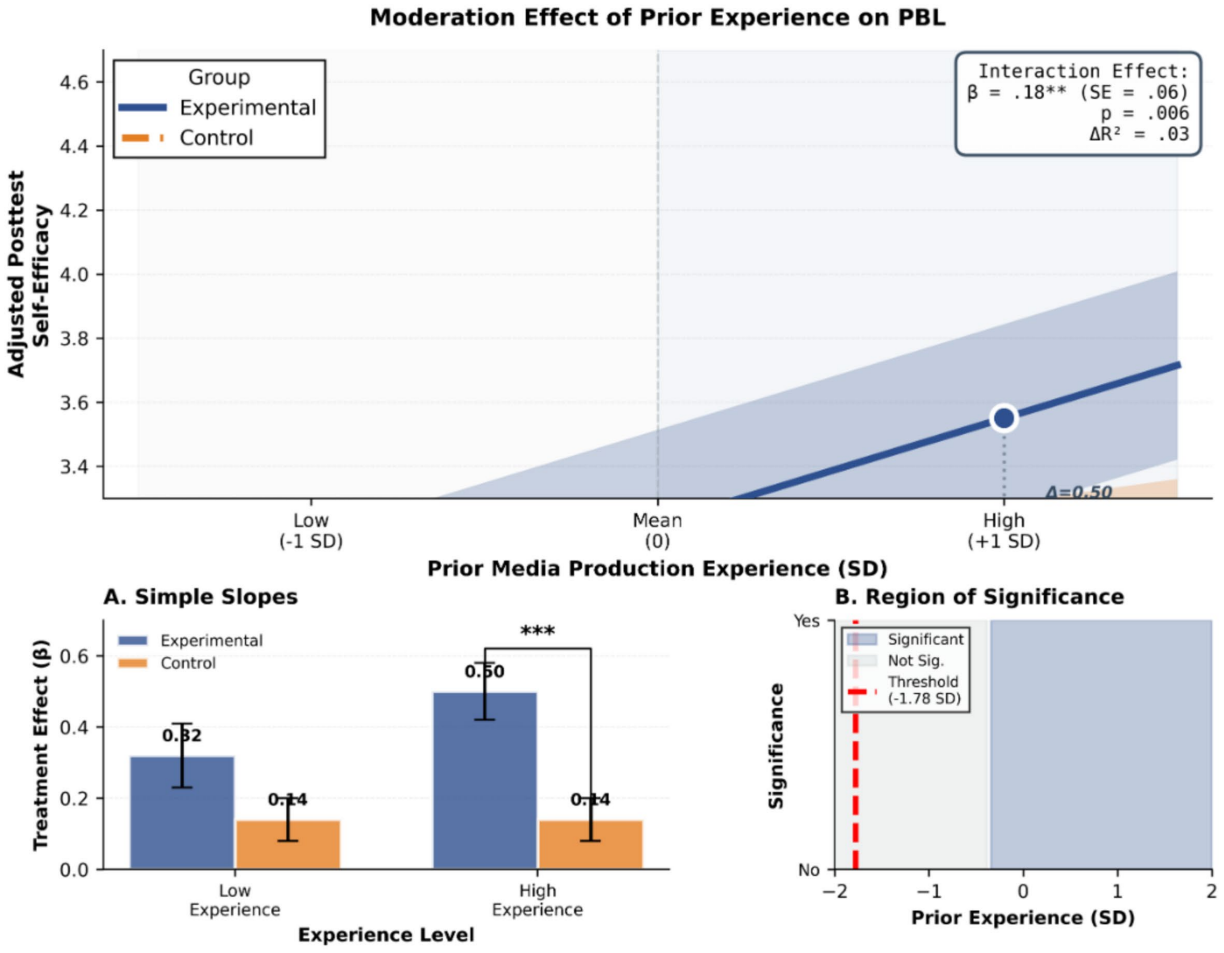
Moderation analysis showing the interaction effect of prior media production experience on the relationship between group assignment (experimental vs. control) and posttest self-efficacy. **(A)** Adjusted posttest self-efficacy by prior experience, with shaded regions representing the experimental and control groups. Inset reports the interaction effect (β = 0.18, SE = 0.06, *p* = 0.006, Δ*R*² = 0.03). **(B)** Left: Bar graph showing that the experimental group outperforms the control group at both low and high prior experience levels, with a significant difference at high experience (β = 0.50, ***p < 0.001). Right: Plot showing the region of significance across levels of prior experience, with a threshold at -1.78 standard deviations.

To explore whether prior media production experience moderates the effect of project-based learning, the researchers used hierarchical regression analysis. Taking the post-test media production self-efficacy as the dependent variable, the first step was to enter the pre-test score and group (dummy variable coding: 0 = control group, 1 = experimental group), the second step was to enter the centralized prior media production experience, and the third step was to enter the interaction term between group and prior experience (Table 11; Figure 5).

**Table 11:** Results of stepwise regression analysis of the impact of project-based learning on media production self-efficacy.

Predictor variable	Step 1 *β* (SE)	Step 2 *β* (SE)	Step 3 *β* (SE)
Pretest self-efficacy score	0.51*** (0.07)	0.50*** (0.07)	0.49*** (0.06)
Group (0 = Control, 1 = Experimental)	0.33*** (0.09)	0.32*** (0.09)	0.32*** (0.08)
Prior media production experience (centered)		0.15* (0.07)	0.15* (0.07)
Group × Prior experience			0.18** (0.06)
*R* ^2^	0.44	0.46	0.49
Adjusted *R*^2^	0.43	0.44	0.47
Δ*R*^2^	0.44***	0.02*	0.03**
*F* for Δ *R*^2^	45.68***	4.53*	7.12**
*F* for model	45.68*** (2, 117)	33.52*** (3, 116)	28.09*** (4, 115)

Please note: *p < 0.05, **p < 0.01, ***p < 0.001.

The interaction term was significant (*β* = 0.183, *p* < 0.01), indicating that prior media production experience has a moderating effect on the effect of project-based learning. The simple slope test further revealed that for those with high prior experience (+1SD), the effect of project-based learning was more significant (*β* = 0.506, *p* < 0.001); while for those with low prior experience (-1SD), the effect of project-based learning was relatively weak but still significant (*β* = 0.140, *p* < 0.05).

This result partially supports H4: Prior media production experience positively moderates the impact of project-based learning on media production self-efficacy, but even for those with low experience, project-based learning still has a significant effect.

### 4.6 Results of qualitative data analysis

In order to explore the mechanism of the effectiveness of project-based learning in depth, this study collected students’ reflection logs and focus group interview data and used the thematic analysis method proposed by Braun and Clarke (2006). Through this rigorous analysis process, qualitative data revealed the deep impact of project-based learning on students, supplementing the results of quantitative research. The analysis process included a series of systematic steps from familiarizing with the data, generating initial coding, to defining themes, to ensure a deep understanding and reliable interpretation of the data. Two researchers independently coded and measured the consistency between coders using Cohen’s *κ* coefficient, which was 0.837, indicating the high reliability of data analysis.

On this basis, the study distilled three main themes, which, respectively, revealed how project-based learning has a profound impact on students’ learning motivation, professional identity and diversified thinking through real task situations, peer collaboration and reflective practice.

#### 4.6.1 Real task situations stimulate learning motivation

One of the core advantages of project-based learning is that it provides students with real-life task scenarios, which directly stimulates their learning motivation. Students generally report that when they realize that the projects they are involved in will have real impacts, their learning enthusiasm is significantly improved. Specifically, this real-life scenario not only increases students’ involvement in the project, but also encourages them to be more serious and responsible throughout the learning process.

One student wrote in a reflective journal: “When I realized that the program produced by the researchers would actually be broadcast on the campus radio station, I felt both excited and stressed. This made me take every detail more seriously”. (Student A, reflective journal). This feedback clearly shows how the authenticity of the project directly stimulated students’ sense of responsibility and motivation, and increased their investment in each task link. This improvement in learning motivation is not only reflected in the completion of the project, but also has a profound impact on students’ self-efficacy and self-cognition.

#### 4.6.2 Peer collaboration promotes diverse thinking

Project-based learning provides students with a platform to share and exchange different perspectives through teamwork. Students have the opportunity to understand problems from multiple perspectives by working with peers of different backgrounds and abilities, which significantly broadens their thinking and problem-solving strategies. In group work, students not only learn to solve complex problems through cooperation, but also develop a more diversified way of thinking through communication and discussion with others.

During the focus group discussion, one student shared, “Working with classmates from different professional backgrounds allowed me to see multiple perspectives on the same problem. It really broadened my thinking”. (Student B, focus group). This feedback shows that through interaction and cooperation with peers, students’ thinking has been greatly expanded, and they are able to apply more diverse perspectives and methods to think when faced with complex tasks. This kind of cooperation and communication has played a role in promoting deep learning in project-based learning, enabling students to improve their problem-solving skills through continuous interaction and reflection.

#### 4.6.3 Reflective practice deepens professional identity

Another notable feature of project-based learning is its emphasis on reflective practice, which helps students combine theoretical knowledge with practical experience and deepens their sense of professional identity. Through regular reflection activities, students can consciously evaluate their performance, identify their own growth and shortcomings, and thus have a clearer understanding of their future career path.

One student said in an interview: “Every reflection session after the project has allowed me to see my growth and shortcomings more clearly. These experiences have made me more certain that this is the career I want to pursue”. (Student C, focus group). This statement shows the importance of reflective practice in student learning. Through this reflective activity, students can continuously improve their self-awareness and professional identity in project-based learning. This reflection not only helps students consolidate the knowledge they have learned, but also makes them more determined in their future career choices.

#### 4.6.4 Theoretical and practical significance of qualitative findings

These qualitative findings provide a rich supplementary explanation for the quantitative results, revealing the internal mechanism by which project-based learning improves students’ self-efficacy and cognitive flexibility in media production. Specifically, real task situations stimulate students’ learning motivation, peer collaboration promotes diversified thinking, and reflective practice further deepens their professional identity. These findings show that project-based learning not only has a significant impact on the skill level, but also plays a key role in students’ learning attitudes, thinking styles, and professional identity.

For educators, these qualitative results provide valuable insights into how to design and implement more effective project-based learning. By combining real tasks, teamwork and reflective practice, project-based learning can effectively enhance students’ learning enthusiasm and professional identity, and enable them to have stronger coping skills and innovative thinking in their future careers. This provides practical suggestions for the curriculum design of broadcasting and hosting majors, and provides important guidance for teaching practices in this field.

## 5 Discussion

### 5.1 Interpretation and significance of the main findings

The main findings of this study support the effectiveness of project-based learning in improving media production self-efficacy, cognitive flexibility, and cross-platform content creation effectiveness among broadcasting and hosting students. These results have multiple implications.

The significant improvement of project-based learning on media production self-efficacy (partial *η*^2^ = 0.197) indicates that by participating in real media production projects, students can gain direct experience of success, which is consistent with the source of proficiency experience emphasized in Bandura’s (1997) self-efficacy theory. This improvement may promote students’ continued efforts and career development in the media industry in the future.

The significant improvement in cognitive flexibility (partial *η*^2^ = 0.164) reflects the advantage of project-based learning in cultivating students’ ability to adapt to complex and changing environments. This echoes the cognitive flexibility theory proposed by Spiro et al. (1988), that is, in ill-structured fields, the ability to flexibly apply knowledge is required. In the current context of media convergence, this ability is particularly important for future media practitioners.

The improvement in cross-platform content creation efficiency (partial *η*^2^ = 0.144) highlights the potential of project-based learning in cultivating students’ all-media literacy. This result is consistent with the participatory culture and media convergence theory proposed by Jenkins (2006), which emphasizes the ability to create and disseminate content in a multi-platform environment.

### 5.2 Dialog with existing theories

#### 5.2.1 Verification and extension of project-based learning theory

The results of this study validate the applicability of the key elements of project-based learning proposed by Larmer et al. (2015) in media education. In particular, the theme of “real task situations stimulate learning motivation” extracted from the qualitative data analysis confirms the core concept of project-based learning that emphasizes authenticity and student initiative. At the same time, this study extends the theory of project-based learning to the field of media production, providing new empirical support for the application of this theory in professional education.

#### 5.2.2 Contribution to self-efficacy theory

The results of this study not only support the applicability of Bandura’s (1997) self-efficacy theory in media education, but also further explore the moderating role of prior experience in this process. The results show that prior media production experience positively moderates the effect of project-based learning (*β* = 0.183, *p* < 0.01), which enriches researchers ‘understanding of the mechanism of self-efficacy formation. This finding suggests that when designing project-based learning interventions, it may be necessary to consider students’ prior experience level and provide differentiated support for students from different backgrounds.

#### 5.2.3 Application of cognitive flexibility theory in media education

This study applied Spiro et al.’s (1988) cognitive flexibility theory to the field of media education and found that project-based learning can significantly improve students’ cognitive flexibility. This finding highlights the importance of cultivating students’ adaptive thinking in the context of media convergence. The theme of “peer collaboration promotes diverse thinking” in the qualitative data further illustrates how project-based learning can cultivate cognitive flexibility through collaboration and multi-perspective problem solving.

### 5.3 Implications of the research results for media education practice

Based on the results of this study, researchers can provide some targeted suggestions for the practice of media education to promote the curriculum design and teaching effect of broadcasting and hosting majors. First, the curriculum design should be more integrated, and the project-based learning method should be systematically incorporated into the curriculum system, especially in practical majors such as broadcasting and hosting. By designing large-scale media production projects across semesters and courses, students can continuously obtain practical opportunities, so as to better combine theory with practice. This integrated curriculum design can enhance students’ continuity and deep participation in different learning stages, and help them improve their self-efficacy and professional ability in complex projects.

Educators should adopt differentiated teaching strategies according to the backgrounds and prior experiences of different students. The results of the study showed that prior media production experience significantly moderated the effects of project-based learning. Therefore, teachers should provide targeted guidance and support based on students’ different experience levels to ensure that every student can benefit from project-based learning. This personalized support can help improve learning outcomes, especially for students who lack practical experience. Differentiated guidance is particularly important.

As the trend of media convergence accelerates, students need to master the ability to flexibly switch between different platforms. Project design should emphasize cross-platform content creation, encourage students to fully consider the characteristics of different media platforms and audience needs, and thus cultivate their omnimedia thinking. This can not only enhance students’ adaptability, but also improve their creativity and flexibility in actual work.

Reflective practice is also an important part of project-based learning. As revealed by qualitative data, reflective activities help students deepen their understanding of the knowledge they have learned and strengthen their professional identity. Therefore, educators should set up regular reflection sessions during the project process to allow students to identify their own growth and shortcomings in reflection, promote the combination of theory and practice, and thus better promote their professional development.

Industry-university collaboration in the media industry should be further deepened. By establishing close ties with the industry, educators can provide students with more authentic project opportunities and enhance the authenticity and relevance of projects through industry feedback. This will not only help improve students’ practical skills, but also help them better cope with career challenges after graduation.

### 5.4 Study limitations

Although this study has made some meaningful findings, it also has some limitations. First, the sample of the study came from only a single institution, which may limit the generalizability of the research results. Therefore, future research can consider adopting a multi-school joint or cross-regional large sample research design to improve the external validity of the results, so that they can be more widely applicable to different educational backgrounds and cultural environments.

The intervention duration of this study was one semester, which can reflect the short-term effects of project-based learning, but may not fully demonstrate its long-term impact. The accumulation and evolution of the effects of project-based learning in the long term deserve further study, so future studies can consider extending the intervention time and even conducting longitudinal tracking to more comprehensively evaluate the lasting impact of project-based learning.

Although this study used an adapted scale to measure media production self-efficacy and cross-platform content creation efficacy, these tools still have room for further optimization. Future research can develop more sophisticated measurement tools to ensure the accuracy and applicability of the measurement, thereby providing a more reliable evaluation method for subsequent research.

This study mainly relied on self-reported efficacy assessment, which may be affected by social desirability bias. Therefore, future research could consider incorporating objective performance assessments, such as actual media production work ratings, to more comprehensively measure the effectiveness of project-based learning. This method of combining subjective and objective assessments would help provide more comprehensive insights.

### 5.5 Future research directions

Future research should explore the multidimensional impact of project-based learning, especially its long-term effects in media education. First, it will be crucial to conduct longitudinal follow-up research. By observing students from enrollment to graduation, and even in their early careers, it will be possible to more comprehensively evaluate the lasting impact of project-based learning. Short-term experiments alone cannot reveal the long-term shaping of project-based learning on students’ professional skills, creative abilities, and self-efficacy. In-depth longitudinal research will help understand the continuity of its educational effects and its impact on future career development.

There may be significant differences in the cultivation of students’ various abilities in projects of different types and complexity. Future research can conduct comparative analysis on various project types. For example, projects with different content forms such as news reporting, documentary production, and radio programs may have different effects on students’ creative expression, technical ability, and critical thinking. Such comparative studies can not only help optimize project design, but also provide educators with more precise teaching strategy guidance.

Cross-cultural comparative research is also a direction that cannot be ignored. Media production itself is deeply influenced by cultural background, and the applicability and effectiveness of project-based learning in different cultural backgrounds may vary significantly. Therefore, future research can conduct cross-cultural comparisons of project-based learning in different countries or regions to explore how cultural differences affect students’ learning experience and project outcomes, thereby providing a more diverse perspective for education reform in the context of globalization.

The continuous advancement of technology has also brought new opportunities for media education. With the widespread application of cutting-edge technologies such as virtual reality (VR) and augmented reality (AR), future research should explore how to integrate these technologies into project-based learning to enhance students’ innovative ability. The integration of technology and project-based learning can not only enrich students’ learning experience, but also provide a new way to cultivate their competitiveness in the future digital media industry.

The role of teachers is particularly critical in project-based learning. How to effectively train teachers to implement this teaching method is an important direction for future research. Project-based learning requires teachers to have higher project design capabilities, guidance skills, and scientific evaluation methods. Therefore, it is crucial to study how to provide systematic training support for teachers. By exploring the best practices of teacher training, future research can provide strong support for improving the teaching quality of project-based learning, thereby promoting the wider application of this teaching method in media education.

## 6 Conclusion

### 6.1 Summary of main conclusions of the study

This study systematically explored the impact of project-based learning on cognitive flexibility and cross-platform content creation effectiveness of students majoring in broadcasting and hosting through a quasi-experimental design.

Project-based learning significantly improved students’ media production self-efficacy [*F* (1, 117) = 28.637, *p* < 0.001, partial *η*^2^ = 0.197]. By participating in real media production projects, students can not only accumulate relevant skills, but also build confidence in their abilities in the process. This result shows that practical tasks have a strong role in promoting students’ self-efficacy.

Project-based learning effectively enhances students’ cognitive flexibility [*F* (1, 117) = 22.945, *p* < 0.001, partial *η*^2^ = 0.164]. Cognitive flexibility is a key ability for students to adapt and solve problems in a changing and complex media environment. This finding reveals that project-based learning can help students improve their flexibility and adaptability in thinking when facing diversified challenges.

Students’ cross-platform content creation efficiency was also significantly improved [*F* (1, 117) = 19.726, *p* < 0.001, partial *η*^2^ = 0.144]. This result highlights the great potential of project-based learning in cultivating students’ cross-platform media production capabilities, omnimedia literacy, and technology integration capabilities, and provides strong support for promoting the cultivation of cross-platform media talents.

The study also found that students’ prior media production experience had a positive moderating effect on the effect of project-based learning (*β* = 0.183, *p* < 0.01). This suggests that when implementing project-based learning, educators need to fully consider students’ background differences, especially their previous practical experience, and provide differentiated support and guidance to ensure that every student can benefit from it and maximize their skills.

Through qualitative analysis, the study revealed three key mechanisms of project-based learning: real task situations can effectively stimulate students’ learning motivation; peer cooperation provides opportunities for diversified thinking, promoting students’ critical thinking and problem-solving skills; reflective practice deepens students’ professional identity. These mechanisms provide deep insights into the effectiveness of project-based learning in education and help understand the deep-seated reasons why it improves students’ multi-faceted abilities.

### 6.2 Theoretical contributions

This study makes the following contributions to related theoretical fields:

This study expands the application of project-based learning theory in the field of media education. Through empirical analysis, the research results verify the effectiveness of the core principles of project-based learning in cultivating media professionals, and further prove the applicability of this theory in professional education. This provides a solid theoretical basis and empirical support for the promotion of project-based learning in media education.

This study enriches the understanding of self-efficacy theory in the context of media education. By exploring the formation mechanism of media production self-efficacy, this study verifies the applicability of Bandura’s self-efficacy theory in the field of media production and further explores the influence of previous media production experience as a moderating variable. This finding deepens the understanding of the formation and development of self-efficacy and expands new perspectives for the application of this theory in specific application fields.

The study combines cognitive flexibility theory with the context of media convergence, revealing the significant role of project-based learning in improving students’ cognitive flexibility. By improving cognitive flexibility, students can better cope with the complex challenges in the media convergence environment, which provides strong support for the application of cognitive flexibility theory in practice and provides a theoretical basis for the future training of talents in a changing media environment.

This study proposed and explored the new concept of cross-platform content creation effectiveness and analyzed its relationship with project-based learning. The introduction of cross-platform content creation effectiveness provides a new perspective for understanding content creation capabilities in the new media environment, broadens the understanding of omnimedia talent training, and fills the gap in existing research.

### 6.3 Practical significance

The findings of this study have had a profound impact on media education practice, especially in curriculum reform, innovation of teaching methods, differentiated teaching strategies, cultivation of all-media talents, promotion of industry-university cooperation, and teacher training, showing outstanding application value and theoretical contribution.

The research provides solid empirical support for the curriculum reform of broadcasting and hosting majors, and strongly advocates the systematic integration of project-based learning teaching methods into the curriculum system. This method breaks the shackles of traditional teaching and emphasizes giving student’s opportunities for continuous practice through real project design across semesters and courses. As Dewey said, “education is life, life is education”, the essence of project-based learning lies in the close integration of theory and practice, so that students can gain a profound learning experience in a dynamic and complex media environment, thereby cultivating compound talents with innovative and practical abilities.

The project-based learning effect mechanism revealed by the study provides a clear direction for teachers to optimize their teaching methods. Teachers should not only focus on designing real task situations, but also promote peer cooperation and strengthen reflective practice, so that students can constantly reflect and adjust their learning strategies under the drive of real tasks, and ultimately achieve the effect of deep learning. This process is in line with Piaget’s understanding of cognitive development, that is, “wisdom lies not only in the accumulation of knowledge, but also in the application of knowledge in ever-changing situations”.

In terms of differentiated teaching strategies, this study found that previous media production experience played a significant moderating role in the effectiveness of project-based learning. This insight provides important inspiration for educators. In the process of teaching implementation, differentiated support and guidance should be tailored according to students’ different backgrounds and experience levels to ensure that each student can benefit the most from project-based learning. This strategy coincides with Vygotsky’s “zone of proximal development” theory, that is, with appropriate support, students can complete more complex tasks than when they learn independently, thereby achieving optimal development results.

The study also highlights the criticality of cross-platform content creation effectiveness, which opens up a new path for the cultivation of omnimedia talents. In the era of media convergence, the cultivation of omnimedia thinking has become an indispensable part. As McLuhan said, “the medium is the message”. Cultivating students’ cross-platform content creation capabilities is not only an improvement in their technical capabilities, but also a profound shaping of their media literacy and innovative thinking. In project design, educators should encourage students to have a deep understanding of the characteristics of different media platforms and cultivate their ability to respond flexibly and express innovatively in a diversified media environment.

The research results provide strong support for promoting in-depth cooperation between universities and the media industry. By strengthening industry-university cooperation and providing students with real project opportunities and industry feedback, the authenticity and relevance of teaching can be greatly enhanced, ensuring that students have the practical ability to “get in touch with the ground before leaving campus” when they enter the workplace. This kind of cooperation not only shortens the distance between academia and practice, but also provides valuable experience for cultivating compound talents with industry acumen and practical operation capabilities.

This has put forward new requirements for teacher training. Teachers not only need to have the ability to design effective projects, but also need to master key skills such as how to guide students to conduct reflective practice and how to evaluate their cross-platform content creation capabilities. It can be said that teachers themselves also need to become active participants and guides in project-based learning through continuous learning and reflection. As Foucault said, “knowledge and power are interdependent”. By mastering effective teaching strategies, teachers can not only guide students to the peak of knowledge, but also shape their future professional paths to a certain extent.

## Data Availability

The original contributions presented in the study are included in the article/supplementary material, further inquiries can be directed to the corresponding author.
